# Ports Digitalization Level Evaluation

**DOI:** 10.3390/s21186134

**Published:** 2021-09-13

**Authors:** Vytautas Paulauskas, Ludmiła Filina-Dawidowicz, Donatas Paulauskas

**Affiliations:** 1 Klaipeda Shipping Research Centre, Marine Engineering Department, Klaipeda University, V. Berbomo Str. 7-5, LT-92219 Klaipeda, Lithuania; vytautaskltc@gmail.com; 2 Faculty of Maritime Technology and Transport, West Pomeranian University of Technology, Szczecin, Ave. Piastów 41, 71-065 Szczecin, Poland; 3 Marine Engineering Department, Klaipeda University, H. Manto g. 84, LT-92294 Klaipeda, Lithuania; paulauskasd75@gmail.com

**Keywords:** seaport, digitalization, digitalization level, accuracy, maritime transport, digital index for ports

## Abstract

Currently, seaports are actively searching for methods and ways to improve their operational efficiency. Digitalization is considered as one of the main directions of current ports’ development. Ports’ digitalization levels are varied and may depend on different factors, including port size, traditions, turnover and handled cargo type, etc. Ports often face decision-making challenges related to assessment of their digitization level and choice of development directions. The article aims to develop a methodology to evaluate ports’ digitalization level. A marketing research tool was used to collect the data needed for the analysis. A mathematical model allowing simulations is proposed and a case study of 30 ports located in the Baltic, North and Mediterranean Seas regions is explored. Based on conducted calculations, a ranking of analysed ports considering their digitalization level was created. The ports were compared within groups of small, medium-sized and large ports. It was estimated that the digitalization level in small and medium-sized ports is about 30% lower than the level of large seaports. The research results may be of interest to seaports striving to assess their level of digitalization and choose the best digital improvement solutions.

## 1. Introduction

Nowadays, one of the essential directions of maritime transport and especially port development is digitalisation and IT systems implementation [1,2]. Different areas of ports’ functioning are covered by digital transformation [3,4], such as port business, management and planning of activities, commercial and supporting services, contact with clients, navigation, etc. Implementation of digital solutions is essential and allows ports and other maritime sectors to increase their efficiency and sustainability, decrease costs and performance time of selected operations, improve information flow and decision-making, reduce paper documents in operational processes in relation to sustainability policy, increase safety, decrease the negative impact of maritime transport on the environment in ports and port areas, enhance innovation, etc. [5,6,7].

Seaports constitute the key nodes of the sea–land transport chains and closer integration into supply chains has a positive effect on their performance [8,9]. Therefore, the benefits of port digitalisation are also essential for the whole supply chain’s performance improvement [10]. Different types of IT system are currently implemented in seaports, both individual solutions and those integrated into complex IT architecture [11].

However, it should be noticed that the digitalisation level of ports is different. Port efficiency measurement systems have been developed [12,13]. While planning further development, seaports are searching for practices already verified by other ports that could be implemented to improve ports’ functioning and sea–land transport operation. Such improvements often deal with maritime transport safety and ports’ aspiration to attract cargo flows and customers, promote port services, and introduce other important amenities for existing and potential cargo owners and shipping companies [14,15,16]. Attention should be paid to the fact that different ports may face challenges in implementing and keeping an appropriate digitalization level that may be influenced by a number of factors, including limited financial, technical and human resources [17].

Evaluation of digitalization level of ports as nodes of sea–land transport chains may be helpful in finding rational solutions for digital systems development. It also gives the opportunity to compare ports’ operation and use evaluation analysis to select the ports with best practices that may be implemented in other ports [18,19,20]. Such approach may be applied by seaports in planning digital systems development, including small and medium-sized ports [21].

The aim of the presented article is to develop a methodology to evaluate the level of port’s digitalization, which may be used by different seaports. The research is conducted within INTERREG project Connect2SmallPorts [22] and presents a continuation of studies already undertaken within this project [17,23,24].

The article includes a literature review section investigating current literature related to the analysed topic. The methodology section describes the method used to conduct the investigations. The case study description, as well as results and their analysis, are reported in the Results section. In order to sum up the research, the discussion and conclusions section concludes.

## 2. Literature Review

The literature review was carried out on the basis of current literature available in the scientific databases, such as Scopus and Web of Science (up to 25 June 2021). The following keywords were used: “digitalization”, “seaport”, “port efficiency”, “efficiency measurement”. The desk research was also supplemented by items found in other available databases.

On the basis of the conducted literature analysis, it can be stated that the issues related to Industry 4.0 and digitalization are often analysed in recent scientific studies [25,26]. Challenges and trends for future research on Industry 4.0 are discussed [27]. Attention is paid to fact that ports are constantly developing and interact with Industry 4.0 in four different modules, namely Port Community Systems (PCSs), cyber-physical systems, Internet of Things (IoT) and Big Data [28].

The necessity of raising the maritime transport industry to the same level of digitalization as other industries has been highlighted [29]. It was also emphasized that not only ports, but also their customers, have to strive to digitalize and optimize their business processes [30].

Digital solutions implementation covers not only seaports, but the whole supply chain operation [7]. Notteboom et al. [31] proposed a typology of fields of activity to pursue green supply chain management that includes five groups of actions, i.e., green shipping, green port development and operations, green inland logistics, seaports and the circular economy, as well as actions in the field of knowledge development and information sharing. Operational supply chain challenges can be explained by technological differences in the available IT applications, which hinder integration [32].

Digitalization is connected with a number of benefits for the company’s horizontal and vertical value chains, as well as with building a digital product and service portfolio [33]. This is also associated with a systematic increase in the flexibility of products and processes through automation, extensive networking and decentralized control mechanisms [34]. It was emphasized that IT technologies facilitate operational decision-making in the transport chains [8]. Additionally, investment in digitalization improve overall information sharing, coordination and visibility capabilities, and performance of supply chains [35].

In the available literature attention is also paid to the technological development of ports. The issues of electrification, digitalization, onshore power supply applications, and energy storage systems are addressed, as well as the energy efficiency of modern ports, including port smart microgrids [36]. Agatić and Kolanović investigated quality factors and opportunities for improving service quality based on the analysis of digital technologies implemented in seaports [37]. Moreover, processes were analysed regarding their degree of automation and digitalization and networking among themselves [34]. Based on this literature analysis, it could be stated that IT technologies and digital solutions are implemented within different areas of port operation.

Heilig et al. [1] provided an overview of the development and state-of-the-art of digital transformation in modern seaports in order to identify current potential and barriers. They presented a conceptual game theoretical framework that allows benefits and cost allocations considering inter-, intra-, and meta-organizational perspectives. Del Giudice et al. [38] also conducted a systematic literature review of digitalization and new technologies in the shipping and seaport industry, highlighting the key variables that play a significant role in achieving the Sustainable Development Goals. Shipping and seaport business models that can meet environmental, economic and social goals through the digitalization of operational processes in the ship–port interface were analysed.

In the reviewed research studies various digital domains have been indicated such as autonomous vehicles and robotics, artificial intelligence, Big Data, virtual reality, augmented and mixed reality, IoT, the cloud and edge computing, digital security, 3D printing and additive engineering [29].

Inkinen et al. [39] identified drivers and technologies that are significant for port digitalization. These drivers were discussed in the context of three alternative scenarios: digital supremacy, business as usual, and digital failure. Among the main drivers, authoritative collaboration (governing driver), logistical hubs and market development (economic driver), port impact in society (societal driver), carbon neutrality (environmental driver), technological trajectories in 3D printing, AI & Big Data, blockchain, IoT and sensor technologies, robotics and automation, laser scaling, and cyber-security were distinguished.

Furthermore, research studies have investigated the role of particular digital solutions in seaport operation. Blockchain technology implementation was considered as a tool for capabilities acceleration within a Smart Ports network [40,41]. Such technology guarantees accountability, simplifies the monitoring process, and accelerates bureaucratic processes and port transactions [42]. It was also stated that shipping and maritime logistics would largely benefit from Big Data, as well as the emerging digital technologies [5].

IoT systems have been proposed to optimize, manage and monitor container transport operations along an intermodal corridor, combining rail scheduling and inland vessel navigation [43]. Such systems may have a modular design, enabling the interaction with external systems, independently of their nature. Henesey et al. [44] suggested an integrated approach to build upon IoT sensors in combination with the lightweight version of a blockchain to improve balance indicators on the trim of a RoPax vessel, as well as to develop appropriate simulation. The described simulation indicated that this approach results in an improvement of 50–160% on current load planning operations for RoPax vessels.

Moreover, the role and importance of PCSs was emphasized [15,30,45], and the National Single Window concept and its impact on sustainability in maritime transport and seaports was stressed [46]. It was indicated that PCSs not only have a positive influence on the adoption of mandatory regulation, but also communication channels, compatibility and infrastructure are key variables to be managed during implementation [47].

The need to improve the efficiency of seaports through digital transformation and interaction of subjects within the new digital space was also highlighted [48]. Digital data (raw data) and digital technologies (including both software platforms and hardware solutions) were addressed [2]. Among applied applications and solutions, location-based identification (e.g., real-time traffic monitoring), spatial map visualizations, and monitoring of traffic and modes of transport are mentioned.

It should be noted that the digitalization level of a port is significantly related to port type and to management by the port authority. The main models, based upon the respective responsibility of the public and private sectors in port management, include [49]: public service ports, tool ports, landlord ports, and corporatized and private service ports. Other types of port could also be defined, considering port traditions, political decisions and the economic environment, etc.

Different kinds of digital system are observed in seaports, including [17]:

- Simple digital systems (e.g., IT systems devoted to specific cargoes) that are not integrated with systems used by the port’s customers and other participants in the supply chains [34,50,51]. Such digital solutions can be established using common programmes (e.g., Excel) and can facilitate the gathering of evidence and evaluation of cargos handling volumes or passenger flows, accounting requirements, etc. Such systems are implemented in selected small and medium-sized ports.
- Intermediate digital systems that are based on block schemes and may be connected with information systems of other entities, e.g., customs, border control, etc. Such digital systems may be created by a port’s IT staff or special IT companies and are not too expensive [23]. Such systems may be applied in small, medium-sized and large ports.
- High precision (modern) digital systems may integrate port terminals with port administration, control bodies, customers and other entities involved in supply chain operations, increasing navigational safety and security. Such modern IT systems are usually developed for the needs of the specific maritime transport or port and other groups of users [12,52]. Such systems may be observed in selected large seaports.

Depending on the port type, digital technology implementation could be provided as an integrated system, as takes place in service or private ports. In landlord and tool ports digitalization may be implemented in several ways, depending on the responsibility taken by important decision-makers. Port authorities mainly focus on digitalization of general issues, such as navigation, port maps, ships’ location in port, actual depths in port, etc. In turn, terminal operators are interested in cargo operations digitalization, such as monitoring of cargo quantity and quality at terminal, stevedoring operations, etc.

Attention is also paid to port operations considering their size [53]. The role of small and medium-sized ports in enhancing the competitiveness and logistics performance of multi-port gateway regions and associated inland logistics systems was analysed by Feng and Notteboom [21,54]. Different variables were considered, such as: (a) cargo volume and market share; (b) international connectivity; (c) relative cluster position; (d) port city and hinterland connection; and (e) logistics and distribution function. Moreover, Philipp [23] stated that smaller ports have no or limited knowledge of Industry 4.0, IoT and blockchain, and what potential they may have. Therefore, it is essential to bring this knowledge to these ports while they are working on their development strategies [55,56].

Digital solutions should also consider operations performed at ports and information flow, e.g., between ship, port and other users of the supply chains [57]. The collection of information becoming automatic, to facilitate real-time decision-making and potentially improve access management for the involved actors, was highlighted [56]. Inter-organizational information systems functioning within the maritime transport chains have been analysed [58], and factors influencing the successful adoption of these systems in seaports have been identified [59]. Heilig and Voß presented a classification and a comprehensive survey of information systems and related information technologies applied in ports [60].

In the recent literature, attention has also been paid to procedures and documents flow. The current regulatory documents in the field of digitalization of seaports have been discussed [61]. Moreover, PCSs and the blockchain scenario for document coordination were compared using mixed methods, incorporating theoretical framework and extensive data from qualitative interviews that were conducted with key actors of the maritime industry [62]. A blockchain scenario for shipping document handling has been developed as an exact solution to issues that PCS was intended to solve: speed-up of communication, data security, elimination of the role of central gatekeeper, establishing point-to-point communication with transactions visibility, and permissioned transparency.

Garibin and Ol’Khovik emphasized the problem of adapting existing buildings and designed documentation to develop models of operation [63]. They proposed a BIM model and the transfer of information in digital form for its repeated use at different stages of the life cycle of the seaport, as well as individual information schemes and elements for standardization of the design of offshore mooring structures, considering turnover in object-oriented format with attribute data.

The impact of digitalization on the sustainability of seaports and maritime transport was analysed by Gonzalez et al. [64]. The authors developed an improved business model and business logic that allows for the rational use of resources and reduces CO_2_ emission and pressure on the environment.

Available publications present the assessment of ports’ efficiency and measure their performance [52,65]. Brooks [66] conducted a comprehensive review of the literature on performance measurement, both at the company level (public and private) and at the program level and explored what constructs may be suitable to measure the performance of devolution programs for ports from a strategic management perspective. El Imran and Babounia [19] stated that port efficiency is the measure of the amount of input and output and their ratio, and is not solely dependent on port performance.

It should be mentioned that Key Performance Indicators (KPIs) are widely applied in shipping marketing and companies’ performance evaluation [67]. KPIs have been developed using Big Data architecture functionalities [68], showing a dashboard to allow easy interpretability of results for planning vessel operations. Marlow and Casaca [13] proposed new port measurement indicators that, besides considering quantitative aspects, also focus on qualitative issues, measure lean port performance and sustain the subsequent development of agile ports. They paid attention to the fact that these indications should bring increasing visibility within the port environment and within the transport chain.

Cullinane et al. [69] evaluated the efficiency of the world’s most important container ports and terminals using the two alternative techniques of Data Envelopment Analysis and the Free Disposal Hull model. It was stated that the availability of panel data, rather than cross-sectional data, would greatly improve the validity of the efficiency estimates derived from all the mathematical programming techniques applied. In turn, Talley [14] noticed that port’s economic performance may also be evaluated by comparing the actual values of its performance indicators to their standards. This author also stated that performance may be assessed from the standpoint of technical efficiency, cost efficiency and effectiveness by comparing the port’s actual throughput with its economically and technically efficient, cost efficient and effectiveness optimum throughput, respectively. However, it should be noted that the mentioned indicators are not related to investigation of ports’ digitalization levels.

Different methods have been proposed to analyse and compare seaports operation efficiency. A method for assessing the significance of the seaport business processes for achieving goals from the standpoint of their further optimization was developed by Bagirov et al. [70]. A benchmarking approach is often used by seaports while developing operational and financial performance to derive useful insights in order to improve their functioning [17,18,19]. For example, this method was used to investigate the current position of the Port of Rijeka (hereinafter Rijeka) in relation to the container business [20]. Marketing research methods are widely used to collect the data needed for in-depth analysis [67].

Recommendations and concrete measures to achieve digitalization and connectivity have been proposed [63,65,71]. Fruth and Teuteberg [5] noticed that there is still a lack of theoretical and empirical work, as well as explanatory approaches to appropriate recommendations for action and restructuring in the area of digitalization in maritime logistics.

An attempt to assess the level of digitalization in ports was made by Paulauskas et al. [17]. Moreover, Philipp proposed to apply digital readiness index for ports (DRIP) and applied this to five selected seaports [23]. A digital auditing tool to discover the digital status of small and medium-sized seaports has been proposed [24]. We continue the research presented in these studies and would like to develop a methodology that would give the opportunity for detailed analysis of the digitalization level of seaports.

On the basis of the conducted literature review, issues related to evaluation of digitalization level of ports have been analysed only to a small extent and there is a lack of studies showing a methodology to evaluate the digitalization level of different kinds of ports. There is still a need to develop this research area and provide decision-making tools to facilitate digital solutions implementation in seaport operation. Therefore, it is necessary to fill this gap and develop the appropriate methodology.

## 3. Materials and Methods

**Figure 1:**
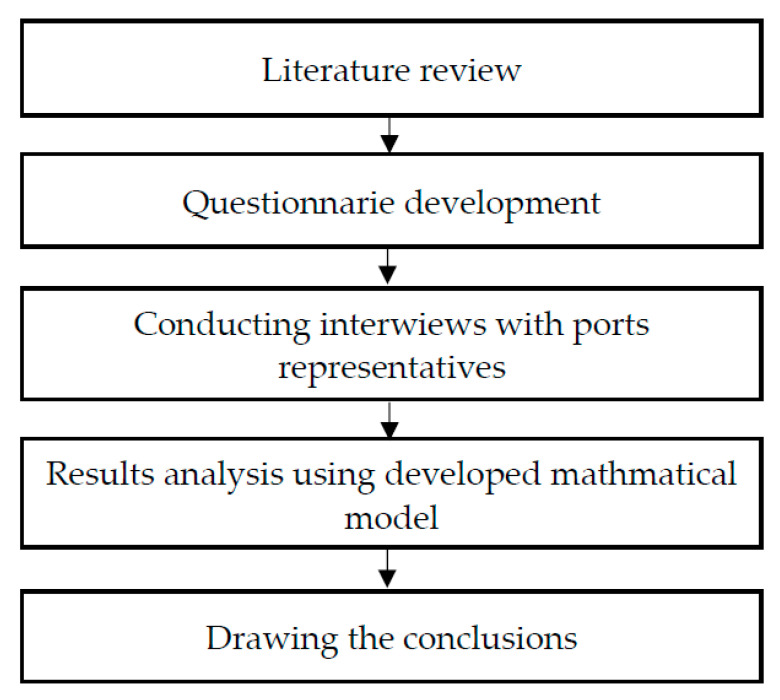
Research methodology.

The methodology applied to conduct the research is shown in Figure 1.

On the basis of the collected information analysis, it was possible to systemize this and discover the strengths, weaknesses, opportunities and threats (SWOT analysis) of the digitalization level of ports or terminals (Table 1). Considering the results of the analysis, the factors that significantly impact the digitalization level of ports, as well as possible prospects, development directions and threats, have been identified.

Within the developed methodology, it was proposed to apply a digital index for ports (DIP) to evaluate their digitalisation level. This index is based on selected factors groups’ analysis. Each factors’ group includes selected activities and parameters. The following groups of factors have been distinguished:

- Scoring group 1 (SG1): navigation (v1); port surface (ports maps) (v2); ships location in port (v3); cargo type in ports, especially dangerous goods (v4).
- Scoring group 2 (SG2): people entering the port, according to ISPS code or terminals’ technology requirements (v5); emergency procedures in port (v6); ETA and ATA of ships (v7); real (actual) depths in port (v8); legal documents valid in the port (e.g., port rules, navigational regulations, etc.) (v9); public procurement issues (v10); port annual reports (v11).
- Scoring group 3 (SG3): port statistical data (v12); port development programs (v13); port development projects (v14); port newsletters (v15); companies operating in port and their activities (v15); technology (v16); port promotion materials (e.g., video, audio) (v17).
- Scoring group 4 (SG4): port organization (v18); port administration working time (v19); additional services in port (v20); port dues and tariffs (v21), human factor (v22).

The evaluation of ports’ digitalization level may be carried out using marketing research tools. For this purpose, a questionnaire was developed and distributed among selected seaport representatives. The questionnaire included 22 factors assigned to four scoring groups, as well as general information about analysed seaports. Considering that analysed selected factors were formulated generally and some were not implemented in the analysed ports, the sub-factors related to chosen factors were used to collect the necessary data.

During interviews conducted in ports, particular factors were assessed by ports’ representatives using a Likert scale [72] (e.g., from 1 to 6, where 1 means the factor is not appropriate for the port/is not utilized by port, and 6 means the factor is appropriate for port/is widely implemented). Respondents can express their opinion and indicate the level of occurrence/utilization of the particular factor in port. The interviews may be conducted several times (e.g., two–five) in order to receive reliable information for further evaluation and analysis.

**Figure 2:**
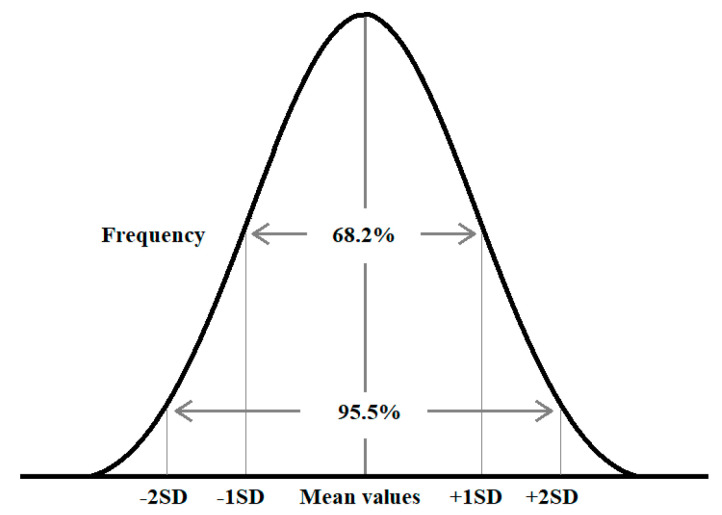
Gaussian distribution showing percentage of values within a certain standard deviation from the mean (own elaboration based on [73]).

The received actual results of interviews may be subjected to mathematical analysis. For this purpose, an accurate mathematical model was developed. It is proposed to base this model on random factors analysis. In case a big number of random factors is analysed, it is possible to use the Lepunov Central Theorem and Normal (Gaussian) distribution [73,74]. Gaussian distribution is a bell-shaped curve, being a type of continuous probability distribution for a real-valued random variable (Figure 2).

It is assumed that during any measurement, values will follow a normal distribution with an equal number of measurements above and below the mean value. The characteristics of Gaussian distributions depend on the Standard Deviation (SD) and may be determined as follows: mean ±1 SD contains probability 68.2% of all values, mean ±2 SD contains probability 95.5% of all values, mean ±3 SD contains probability 99.7% of all values [73].

It should be noted that the described approach was chosen for the analysis of data collected from particular ports, and mean values and standard deviations have to be estimated for these data. It was assumed that for analysis of selected ports’ digitalization level, the standard deviation is ±1 with probability 68.2%. This is due to the fact that the data collected during the interviews may vary.

Evaluation of ports’ digitalization level is based on typical methods of data comparison that can be described using Equation (1):

(1) $$E_{P_{i}}=\frac{1}{\eta_{k}}F_{i},$$

where:

$E_{P_{i}}$—*i* port’s digitalisation level.
$\eta_{k}$—correlation coefficient, assuming that this could vary in a range between 0.96–0.98.
$F_{i}$—assessment of all scoring groups for *i* port, that can be calculated using Equation (2):
  (2) $$F_{i}=\sum_{n=1}^{5}\left(\frac{\sum_{j=1}^{m_{n}}S_{nj}}{m_{n}}k_{S_{nj}}\right),$$
  where:
  n—number of scoring group, *n* = 1, …, 4 (this depends on the selected groups of factors).
  $S_{nj}$—assessment of scoring factor *j* in group *n*, given by respondent.
  $m_{n}$—number of factors in group *n*.
  $k_{S_{nj}}$—weight coefficient of the scoring group *n*.

In case the analysed data were obtained from the same expert (port representative) but at different time periods, the fluctuation and differences in the expert’s opinions may be observed during data comparative analysis. This may be caused by differences in respondent’s perception of the problem area when the same questions are asked, as well as changes taking place within analysed factors. Therefore, data filtration is needed. Filtration of data collected during the interviews can be achieved using a Kalman filter applying the Equation (3) [75]:

(3) $$x_{k}=Ax_{k-1}+Bu_{k}+\omega_{k},$$

with observations $z_{k}$ (Equation (4)):

(4) $$z_{k}=Hx_{k}+\upsilon_{k},$$

where:

*A*, *B*, *H*—coefficients.
*ω_k_*, *υ_k_*—sequence of noisy observations.
*x_k_*, *u_k_*—control vectors.

The appropriate computational model to conduct simulations in order to analyze the interview results has been developed.

The proposed method of digitalization level evaluation is focused on the DIP scoring band analysis. To calculate the size of the random error or the DIP, scoring band, dispersion and/or “maximal distribution” mathematical methods can be used. It was established that the size of the random error (e or $\Delta t_{P}$) in the dispersion method is comparable with dispersion ($\sigma_{y}$) [74,76]. The dispersion method was implemented to evaluate the DIP scoring bands and can be expressed using the Equation (5) [77]:

(5) $$\sigma_{y}^{2}=\frac{1}{l-1}\sum {\left(t_{i}-t_{y}\right)}^{2},$$

where:

l—the number of the measurements (interviews conducted in ports).
$t_{i}$—particular measurement results (port’s DIP scoring).
$t_{y}$—mathematical expectation of the average DIP scores, which can be calculated using Equation (6).
  (6) $$t_{y}=\frac{\sum_{i=1}^{l}t_{i}}{l}.$$

Finally, the DIP scoring band with determined probability (e.g., 63–68%) ($\Delta t_{P}$) can be presented as follows (Equation (7)):

(7) $$e=\Delta t_{P}=\pm \sqrt{\sigma_{y}^{2}}.$$

The DIP scoring band $t_{P}$ is calculated applying Equation (8):

(8) $$t_{P}=t_{y}\pm \Delta t_{P}.$$

Similarly, the DIP scoring band can be calculated using the “maximal distribution” method. For the research, this can be expressed as shown in Equation (9) [74]:

(9) $$t_{P}=t_{y}\pm \;P^{\prime }\cdot \Delta t\cdot k_{t},$$

where:

$P^{\prime }$—probability coefficient (it has been proposed that in case of a probability of 63–68%, the coefficient should equal 1; in the case of a probability of 95%, the probability coefficient should be 2, and in case of a probability of 99.7%, the probability coefficient equals 3).
Δt—difference between maximum and minimum ports’ DIP scoring values.
$k_{t}$—coefficient, which depends on the number of measurements (the number of possessed data): in case the number of data is 3, this coefficient will be 0.55; in case the data number is 4, this coefficient will be 0.47, and similarly depending on the data number 5—0.43; 6—0.395; 7—0.37; 8—0.351; 9—0.337; 10—0.329; 11—0.325; 12—0.322 and so on. The minimum value of this coefficient is about 0.315, in case the number of items of collected data is more than 15.

In order to conduct results analysis, the evaluated ports should be specified and it is important to divide them depending on their DIP score, and port importance in logistics chains and cargo turnovers. This is necessary because ports may have different possibilities and resources for digital solutions implementation.

It was noted that seaports’ operation is varied, considering not only the organisation of their activity, but also their capacity and productivity. In order to conduct detailed results analysis of ports digitalization levels within the presented methodology, it is proposed to divide ports considering the following criteria:

- number of serviced passengers (in case of passenger ports or ferry terminals):
  ○ up to 50,000 passengers per year
  ○ from 50,000 up to 100,000 passengers per year
  ○ from 100,000 up to 1,000,000 passengers per year
  ○ more than 1,000,000 passengers per year
- cargo type (in case of cargo ports or terminals):
  ○ containers
  ○ brake-bulk cargo, e.g., wood products
  ○ bulk cargo, e.g., fertilizers, coal, ore, etc.
  ○ liquid cargo, e.g., crude oil, oil products, LNG, etc.
  ○ mixed cargo
- cargo turnover of ports or terminals:
  ○ small ports (with annual turnover up to 1 million tons)
  ○ medium-sized ports (with turnover from 1 million tons up to 10 million tons per year)
  ○ large ports (with annual turnover of more than 10 million tons)

For example, for the digitalization level evaluations that are conducted for ports located in European region, the seaports may be additionally divided, considering their importance in the Trans-European Transport Network (TEN-T), distinguishing core (TEN-T) ports, comprehensive and Non-TEN-T ports.

It is also proposed to divide seaports considering DIP score range into the following groups of ports [23]:

- analogue (DIP score from 1 up to 2.4)
- monitor (DIP score from 2.5 up to 3.4)
- adopter (DIP score from 3.5 up to 4.4)
- developer (DIP score from 4.5 up to 5.4)
- smart (DIP score from 5.5 up to 6.0) ports

In order to evaluate ports considering their digitalization level and provided activity, it is important to classify ports appropriately using selected criteria, for example, DIP score and port type (considering Core (TEN-T) port, comprehensive port, Non-TEN-T port, port turnover, port location (country), etc.). Other characteristics of ports may also be distinguished. According to the above-mentioned possible division of ports, within the conducted research the ports’ digitalization level will be assessed considering port size based on annual turnover. Therefore, small and medium-sized and large ports are explored separately.

On the basis of results analysis, conclusions are drawn.

## 4. Results

### 4.1. Case Study Description

**Figure 3:**
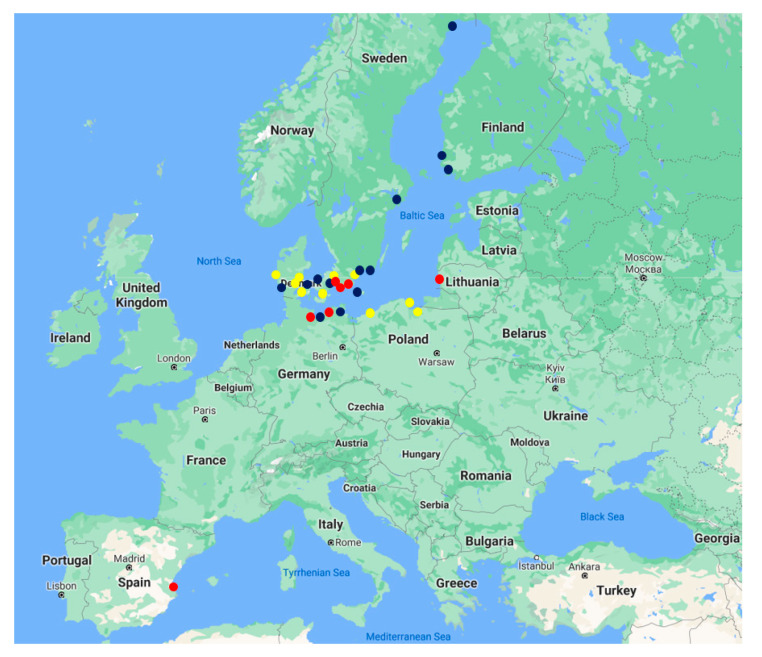
Location of analysed ports: small ports are coloured yellow, medium-sized ports blue and large ports red.

For the case study analysis, 30 seaports were selected that were willing to take part in the research. These seaports are located in the Baltic, North, and Mediterranean Seas (Figure 3). The analysed ports were divided into three groups depending on their cargo turnover: small, medium-sized and large ports. It was assumed that the types of cargo serviced in ports and their volumes impact the decisions related to possible resource allocation into the ports’ digitalization level improvement.

The list of analysed seaports is presented in Table 2. Among these, small ports are designated as S1–S10, medium-sized (M1–M13) and large ports (L1–L7). Most of the ports are located in the Baltic Sea region, but it was also possible to obtain the opinions of ports located in other geographic areas.

The interviews with representatives of these seaports were conducted in 2019 and 2020 during face-to-face meetings in ports. The representatives were asked to fill in the developed questionnaire and give their assessments of the factors influencing digitalization levels using a set measurement scale. The number of conducted interviews was between one and three (depending on the port) and allowed collection of the data necessary to conduct the research. Subsequently, these data were analysed in detail.

An example of part of the questionnaire and the assessments given by a representative of Elblag seaport (Poland) during the first round of interviews is shown in Table 3.

### 4.2. Results Analysis

**Figure 4:**
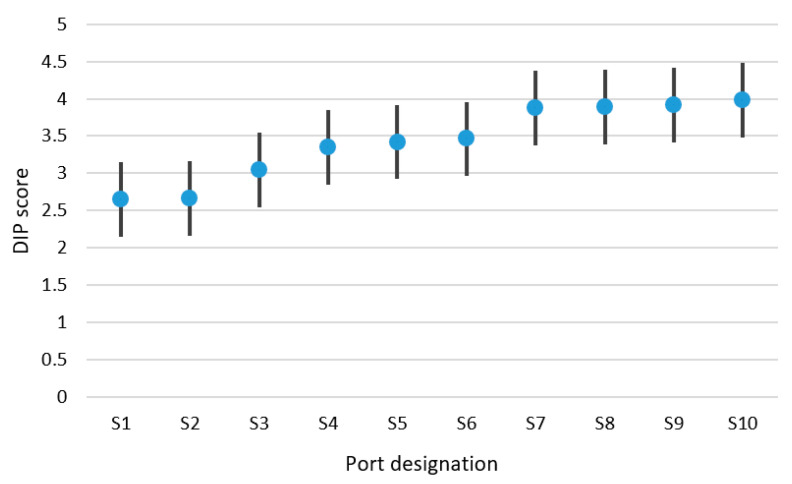
Small ports DIP score and DIP scoring band.

**Figure 5:**
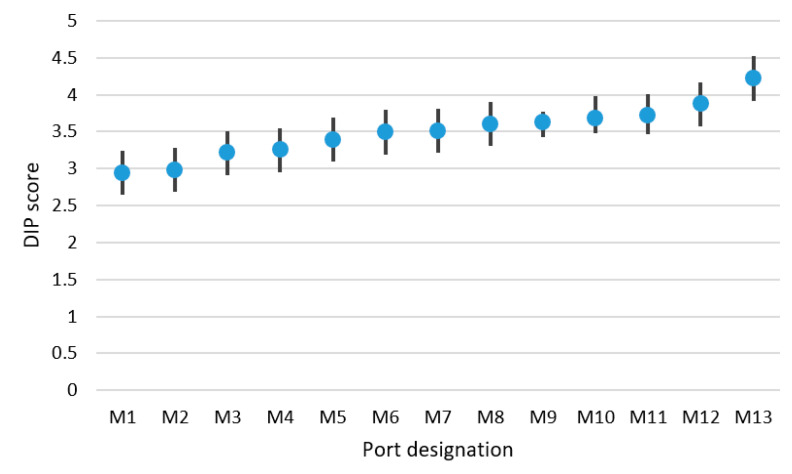
Medium-sized ports DIP score and DIP scoring band.

**Figure 6:**
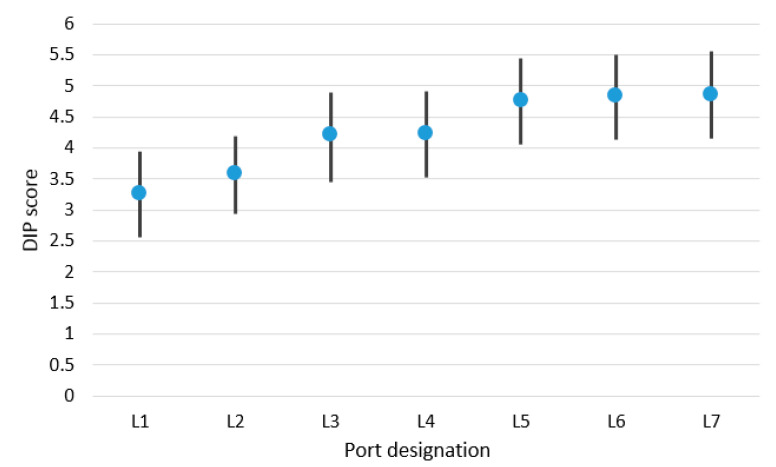
Large ports DIP score and DIP scoring band.

On the basis of data accumulated during the interviews, it was possible to calculate the DIP score for selected analysed ports. DIP filtration of achieved values was carried out using a Kalman filter. The results of DIP score values for the small ports are presented in Table 4, for the medium-sized ports in Table 5 and for the large ports in Table 6. DIP scoring bands were also calculated for groups of small, medium-sized and large ports. These research results are presented in Figure 4, Figure 5 and Figure 6. Designation of ports presented in Figure 4, Figure 5 and Figure 6 matches the designation shown in Table 4, Table 5 and Table 6.

On the basis of calculations conducted using the developed methodology, the mathematical expectation of the DIP score was determined for the small ports group and equalled 3.456. The DIP score band for this group of ports was achieved and constituted 0.506. The accuracy of the DIP score values calculation has been assessed at a level of 14.7%.

The same calculations were conducted for the group of medium-sized ports. The mathematical expectation of DIP score has been obtained and ranged by 3.46. The DIP score band was estimated (0.265), as well as the accuracy of the DIP score values, assessed at a level of 7.7%.

In turn, the estimated mathematical expectation of DIP score for the large ports group equalled 4.24. The DIP score band for this ports group was 0.63, and the accuracy of calculated DIP score values was achieved at a level of 14.9%.

Results analysis revealed that the DIP scores of ports allocated to different groups is varied (Table 7). Differences are observed within port groups, as well as between groups. It should be noted that the digitalization level of small and medium-sized ports is about 30% lower compared to the level assessed for ports assigned to the large ports group.

**Figure 7:**
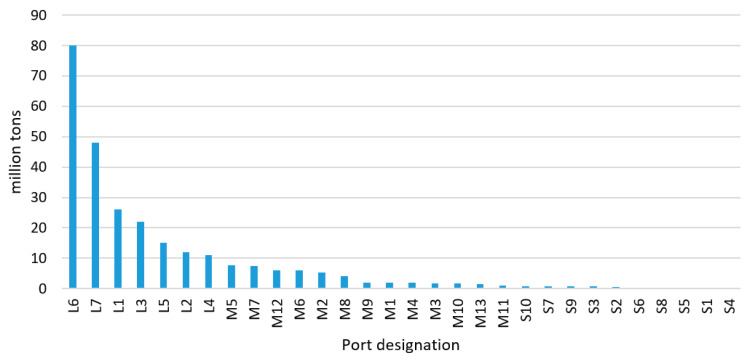
Ranking of analysed ports’ annual turnover.

**Figure 8:**
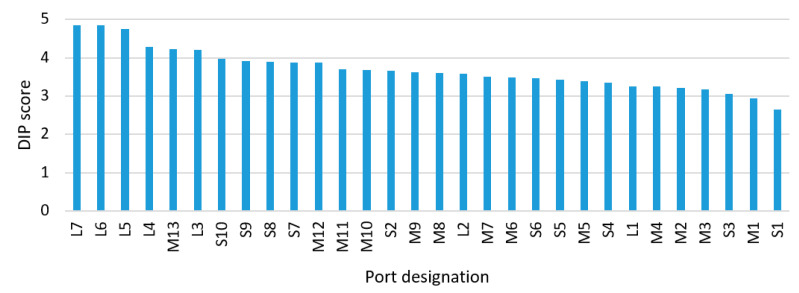
Ranking of analysed ports’ DIP score.

**Figure 9:**
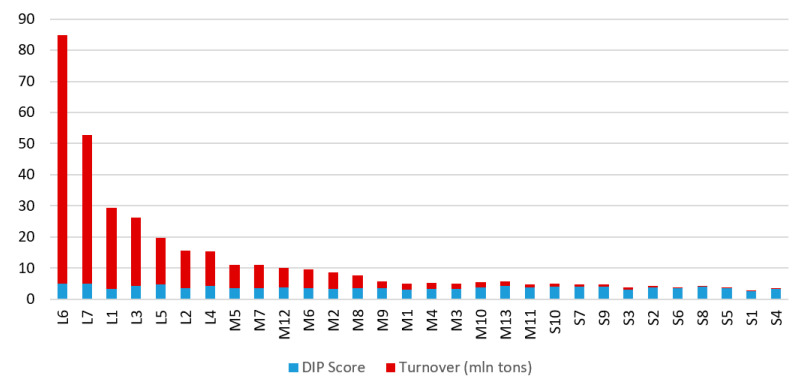
Ranking of analysed ports’ turnover and DIP score.

Moreover, it is necessary to point out that a similar digitalization level of ports does not mean that they have similar cargo turnover. The rankings of analysed ports’ turnover and calculated DIP scores are presented in Figure 7, Figure 8 and Figure 9. For example, Rostock (Germany) has a turnover of more than 25 million tons per year and is classified as a large port. At the same time, Karlskrona’s (Sweden) annual turnover is about 2 million tons, while it is assigned to the medium-sized ports group. In turn, Assen’s (Denmark) turnover is up to 0.02 million tonnes per year (it is placed within the small ports group). Nevertheless, these ports have a similar DIP score and are assigned to the same category of digitalization level.

On the basis of the results analysis it was observed that, within analysed groups of ports, seaports with lower cargo turnover have less possibilities of digitalizing port operations and other activities. This may be due to the limited financial support and volume of investment for digitization.

## 5. Discussion and Conclusions

As the result of the conducted research, a methodology to evaluate the level of a port’s digitalization has been proposed and its verification in the selected case study analysis was carried out. It was proposed to apply a DIP score to assess ports’ digitalisation levels. A mathematical model was proposed for data analysis. Thirty European seaports with different cargo turnovers were evaluated using the developed approach.

Presented methodology could be applied in practice to evaluate ports’ digitalization levels for small, medium-sized and large ports. The results may also be used by ports, terminals or other entities for comparative analysis. The presented approach and implementation makes it possible to assess the current state of ports’ digitalization level which may allow information on the areas of a ports’ activity that need further improvement. Moreover, the proposed approach may be used as a basis for benchmarking and allow ports to choose rational solutions while making decisions related to investments in digitalization development in ports.

It should be mentioned that differences in the digitalisation level of analysed ports have been observed. Calculated small ports average digitalization DIP score (after filtration) was achieved in a range from 2.65 to 3.98, medium-sized ports DIP score was from 2.94 to 4.22 and large ports DIP score from 3.25 to 4.85. This data give rise to the conclusion that small and medium-sized ports’ evaluated digitalization level is lower than the level of large ports. It was estimated that the digitalization level in small and medium-sized ports is about 30% lower compared to the level of large seaports.

On the basis of the developed methodology it was possible to evaluate the digitalisation level of ports using the DIP score. However, it should be mentioned that the achieved results are limited to ports located within the analysed geographical areas, mainly the Baltic, North and Mediterranean Seas. In future studies it would be reasonable to extend the research and analyse the digitalization level of ports located in other regions, e.g., the Black Sea, Tyrrhenian Sea, Adriatic Sea and other regions.

Accuracy of evaluation distributed considering port size (annual turnover) is as follows: large seaports—up to 14.9%, medium-sized ports—up to 7.7%, small ports up to 14.7%. The large fluctuation in the accuracy of evaluation is mainly caused by differences in answers within the questionnaires by particular respondents.

Attention should be paid to the fact that, despite the chosen factors investigated, the digitalization level of ports may be influenced by other internal and external factors. For example, available funds and the economic environment of a port may influence the volume of investments in digitalization and its level. Therefore, detailed analysis and possible extension of factors influencing these ports’ operation may be considered.

The level of technological development of ports so far and the pace of development may also be discussed. It can be difficult to eliminate gaps in ports’ digital development at a rapid pace, and such activities may need time. Therefore, it is reasonable to implement a benchmarking approach to plan future investments in digitalisation.

On the basis of the developed methodology, which allows evaluation of a port’s digitalization level, special software may be developed dedicated to such analysis for different ports in different countries of the world.

It should be noted that new digital solutions are constantly applied to port development which may influence the digitalisation level. Therefore, it is worth continuing the research and to investigate further changes in ports’ digitalization level ranking.

Moreover, on the basis of the analysed case study results it was observed that ports with lower cargo annual turnover have a limited possibility to digitalize port operations and related activities. This fact may be caused by limited funds and organizational facilities for implementation of large-scale investments. Therefore, it could be stated that digitalization of small and medium-sized ports’ activities and management is essential. An increasing digitalization level of these ports could stimulate their activities and increase port service possibilities. This may contribute to the increase of their competitive position in the maritime transport services market, as well as strengthen the operation of different cargo sea–land transport chains.

## Figures and Tables

**Table 1:** SWOT analysis of the digitalization level of ports or terminals.

Strengths	Weaknesses
optimal management; enough human resources; good functionality (IT); modern technology; reliable available information; high productivity; clear and sufficient funds structure; optimal lead-time of ships and cargo in port or terminal, etc.	poor management; lack of human resources; unclear functionality (IT); old technology; inconclusive and few information; low productivity; poor and unclear funds structure; insufficient lead-time of ships and cargo in port or terminal, etc.
**Opportunities**	**Threats**
transport policy development influencing ports’ management improvement; strengthening the position of the maritime transport sector creating potential possibilities to improve and increase human resources in ports; optimization of ports’ functionality (IT) due to development of modern IT solutions applied in transport chains; hinterland infrastructure development affecting new and modern technology implementation in ports; optimistic forecasts for economic growth and an increase in trade that facilitate the need for new and modern information systems implementation; favorable political conditions influencing a rise in ports’ productivity; potential possibilities to increase financing from external sources; possibilities to integrate and organize better the port operation within supply chains, allowing optimal lead-time of ships and goods in port or terminal, etc.	lack of sufficient support of ports’ development and management within the transport policy; decline in ports’ position in the market, influencing less attractiveness for highly qualified staff; changes in functionality (IT) due to cargo flow shifts within transport chains; lack of hinterland infrastructure development within transport chains affecting the limitation of new technology implementation in ports; decrease in cargo volumes transported within transport chains influencing the resources available for port or terminal information systems development; political conflicts and market instability influencing ports’ productivity stagnation; lack of financial support from external sources or economic crisis; disruptions in supply chain integration and operation leading to constant lead-time problems of ships and goods in port or terminal, etc.

**Table 2:** List of seaports analysed in the case study.

Small Ports (S)	Medium-Sized Ports (M)	Large Ports (L)
S1—Hel (Poland), Baltic Sea S2—Landskrona (Sweden), Baltic Sea S3—Vordingborg (Denmark), Baltic Sea S4—Assens (Denmark), Baltic Sea S5—Elblag (Poland), Baltic Sea S6—Kolobrzeg (Poland), Baltic Sea S7—Vejle (Denmark), Baltic Sea S8—Hvide-Sande (Denmark), North Sea S9—Horsens (Denmark), Baltic Sea S10—Sölvesborg (Sweden), Baltic Sea	M1—Kalundborg (Denmark), Baltic Sea M2—Karlshamn (Sweden), Baltic Sea M3—Karlskrona (Sweden), Baltic Sea M4—Koge (Denmark), Baltic Sea M5—Naantali (Finland), Baltic Sea M6—Wismar (Germany), Baltic Sea M7—Lulea (Sweden), Baltic Sea M8—Esbjerg (Denmark), North Sea M9—Stralsund (Germany), Baltic Sea M10—Lindo (Denmark), Baltic Sea M11—Ronne (Denmark), Baltic Sea M12—Rauma (Finland), Baltic Sea M13—Södertälje (Sweden), Baltic Sea	L1—Rostock (Germany), Baltic Sea L2—Ystad (Sweden), Baltic Sea L3—Lubeck (Germany), Baltic Sea L4—Trelleborg (Sweden), Baltic Sea L5—Copenhagen (Denmark)—Malmo (Sweden), Baltic Sea L6—Valencia (Spain), Mediterranean Sea L7—Klaipeda (Lithuania), Baltic Sea

**Table 3:** Part of questionnaire filled by Elblag port representative (first round of interviews).

Factor	Sub-Factor	Abbreviation	Assessment Given by Port Representative (Round 1)	Applied Scale Range
Port development programs	Digitalization strategy	v13-1	3	1—there is no activity in port, 6—the activity is implemented
	Port digitalization development program	v13-2	2	
	Digitalization pilot initiatives	v13-3	2	
	Funds for the port development	v13-4	3	
Technology	Smart Enterprise Resource Planning System	v16-1	3	1—technology is not known, 6—comprehensive usage of the technology
	Smart Warehouse Management System	v16-2	4	
	Smart Port Community System	v16-3	4	
	Web-based Communication Platforms	v16-4	4	
	Mobile Data Access for Employees	v16-5	4	
	Mobile Data Access for Customers	v16-6	4	
	Internet-of-Things	v16-7	5	
	Cloud Computing	v16-8	5	
	Localization Technologies	v16-9	5	
	Sensors	v16-10	5	
	Big Data and Predictive Analytics	v16-11	5	
	Blockchain	v16-12	4	
	Artificial Intelligence	v16-13	2	
	Robotics	v16-14	2	
	Drones	v16-15	2	
	Autonomous Solutions	v16-16	2	
	Digital Twinning, Augmented and Virtual Reality	v16-17	2	
Port promotion materials	Personal Network	v17-1	4	1—very bad, 6—very good
	Printed Media	v17-2	4	
	Internet	v17-3	5	
	Social Media	v17-4	5	
	Fairs	v17-5	4	
	Conferences	v17-6	4	
	Associations and Consultancies	v17-7	4	
	Scientific Institutions	v17-8	3	
Port organization	Port management system	v18-1	1	1—very bad, 6—very good
	IT infrastructure	v18-2	4	
	Automation technology	v18-3	3	
	Data analytics	v18-4	3	
	Data security/communications security	v18-5	4	
	Development/application of assistance systems	v18-6	3	
	Collaboration software	v18-7	3	
	Non-technical skills such	v18-8	4	
	Port diversification programs	v18-9	4	
Human factor	Port management approach to digitalization	v22-2	4	1—very bad, 6—very good
	Port management education level	v22-3	4	
	Personal network system	v22-4	4	
	Ability of port IT staff and readiness to implement digitalization tasks	v22-5	4	
	Port staff periodical training system	v22-6	4	
	Funds for port staff education and training	v22-7	4	

**Table 4:** DIP score values calculated for small ports.

Port	Hel	Lands-Krona	Vording-Borg	Assens	Elblag	Kolobrzeg	Vejle	Hvide-Sande	Horsens	Solves-Borg
	S1	S2	S3	S4	S5	S6	S7	S8	S9	S10
DIP	2.54	2.55	3.08	3.37	3.43	3.47	3.9	3.91	3.99	4.03
Filtrated DIP	2.65	2.66	3.05	3.35	3.42	3.46	3.88	3.89	3.92	3.98

**Table 5:** DIP score values calculated for medium-sized ports.

Port	Kalun-Borg	Karls-Hamn	Karls-Krona	Koge	Naantali	Wismar	Lulea	Esbjerg	Stral-Sund	Lindo	Ronne	Rauma	Soder-Talje
	M1	M2	M3	M4	M5	M6	M7	M8	M9	M10	M11	M12	M13
DIP	2.85	2.92	3.18	3.23	3.4	3.49	3.51	3.6	3.62	3.69	3.73	3.92	4.32
Filtrated DIP	2.94	2.98	3.21	3.25	3.39	3.49	3.51	3.6	3.62	3.68	3.71	3.87	4.22

**Table 6:** DIP score values calculated for large ports.

Port	Rostock	Ystad	Lubeck	Trelleborg	Coppenhagen-Malmo	Valencia	Klaipeda
	L1	L2	L3	L4	L5	L6	L7
DIP	3.15	3.50	4.20	4.22	4.78	4.88	4.90
Filtrated DIP	3.25	3.58	4.20	4.22	4.75	4.84	4.85

**Table 7:** Compilation of analysed ports’ turnover and calculated DIP scores.

Port Designation	Port	Turnover (mln tons)	DIP Score
L6	Valencia (Spain), Mediteraininan Sea	80	4.84
L7	Klaipeda (Lithuania), Baltic Sea	48	4.85
L1	Rostock (Germany), Baltic Sea	26	3.25
L3	Lubeck (Germany), Baltic Sea	22	4.2
L5	Copenhagen (Denmark)–Malmo (Sweden), Baltic Sea	15	4.75
L2	Ystad (Sweden), Baltic Sea	12	3.58
L4	Trelleborg (Sweden), Baltic Sea	11	4.28
M5	Naantali (Finland), Baltic Sea	7.6	3.39
M7	Lulea (Sweden), Baltic Sea	7.5	3.51
M12	Rauma (Finland), Baltic Sea	6.1	3.87
M6	Wismar (Germany), Baltic Sea	6.1	3.49
M2	Karlshamn (Sweden), Baltic Sea	5.3	3.21
M8	Esbjerg (Denmark), North Sea	4.1	3.6
M9	Stralsund (Germany), Baltic Sea	2	3.62
M1	Kalundborg (Denmark), Baltic Sea	2	2.94
M4	Koge (Denmark), Baltic Sea	2	3.25
M3	Karlskrona (Sweden), Baltic Sea	1.8	3.18
M10	Lindo (Denmark), Baltic Sea	1.7	3.68
M13	Sodertalje (Sweden), Baltic Sea	1.5	4.22
M11	Ronne (Denmark), Baltic Sea	1	3.71
S10	Sölvesborg (Sweden), Baltic Sea	0.9	3.98
S7	Vejle (Denmark), Baltic Sea	0.78	3.88
S9	Horsens (Denmark), Baltic Sea	0.75	3.92
S3	Vordingborg (Denmark), Baltic Sea	0.7	3.05
S2	Landskrona (Sweden), Baltic Sea	0.5	3.66
S6	Kolobrzeg (Poland), Baltic Sea	0.3	3.46
S8	Hvide-Sande (Denmark), North Sea	0.15	3.89
S5	Elblag (Poland), Baltic Sea	0.15	3.42
S1	Hel (Poland), Baltic Sea	0.05	2.65
S4	Assens (Denmark), Baltic Sea	0.02	3.35

## Data Availability

Data available on “South Baltic Small Ports as Gateways towards Integrated Sustainable European Transport System and Blue Grown by Smart Connectivity Solutions” project website: https://connect2smallports.eu, accessed on 1 August 2021.
